# Identifying the determinants of response to MDM2 inhibition

**DOI:** 10.18632/oncotarget.3116

**Published:** 2015-02-03

**Authors:** Anne Y. Saiki, Sean Caenepeel, Elissa Cosgrove, Cheng Su, Michael Boedigheimer, Jonathan D. Oliner

**Affiliations:** ^1^ Oncology Research, Amgen, Inc., Thousand Oaks, California, USA; ^2^ Genome Analysis Unit, Amgen, Inc., South San Francisco, California, USA; ^3^ Biostatistics, Amgen, Inc., Seattle, Washington, USA; ^4^ Molecular Sciences, Amgen, Inc., Thousand Oaks, California, USA; ^5^ Department of Molecular Biology and Genetics, Cornell University, Ithaca, New York, USA

**Keywords:** *TP53*, *MDM2*, amplification

## Abstract

Previous reports have provided evidence that p53 mutation is a strong negative predictor of response to MDM2 inhibitors. However, this correlation is not absolute, as many p53^Mutant^ cell lines have been reported to respond to MDM2 inhibition, while many p53^WT^ cell lines have been shown not to respond. To better understand the nature of these exceptions, we screened a panel of 260 cell lines and noted similar discrepancies. However, upon extensive curation of this panel, these apparent exceptions could be eliminated, revealing a perfect correlation between p53 mutational status and MDM2 inhibitor responsiveness. It has been suggested that the *MDM2*-amplified subset of p53^WT^ tumors might be particularly sensitive to MDM2 inhibition. To facilitate clinical testing of this hypothesis, we identified a rationally derived copy number cutoff for assignment of functionally relevant *MDM2* amplification. Applying this cutoff resulted in a pan-cancer *MDM2* amplification rate far lower than previously published.

## INTRODUCTION

The tumor suppressor protein p53 plays a critical role in protecting cells from various stresses, such as DNA damage and hypoxia [1]. In response to these triggers, activated p53 upregulates the transcription of a host of genes involved in cell cycle arrest, apoptosis, DNA repair, and senescence [2]. Tumor cells are under constant cellular stress, and there is a selective survival advantage for such cells to disrupt the p53 pathway. Indeed, inactivation of p53 by mutation and/or loss occurs in approximately 50% of human tumors [2].

MDM2, another key member of the pathway, negatively regulates p53 by 1) binding to and blocking the transcriptional activation domain of p53, 2) exporting p53 from the nucleus to the cytoplasm, and 3) promoting the degradation of p53 through its E3 ubiquitin ligase activity [3]. Gene amplification of *MDM2* occurs at high frequency in sarcomas and at low frequency in cancers of the brain, bladder, stomach, lung, skin, and breast [4].

The MDM2-p53 protein-protein interaction can be disrupted by small molecule inhibitors which occupy the p53 binding pocket of MDM2, leading to the stabilization of p53 and activation of the pathway [5]. Several MDM2 inhibitors are currently in clinical development [6, 7]. In order to better understand which patients might realize the greatest benefit from MDM2 inhibitor treatment, we set out to identify the determinants of sensitivity and/or resistance by screening a broad panel of tumor cell lines. Additionally, we mined data generated by the TCGA Research Network [4] to rationally define parameters for clinical testing of the hypothesis that *MDM2* amplification might enhance sensitivity of p53^WT^ tumors to MDM2 inhibition.

## RESULTS

### Sensitivity profiling of MDM2 inhibitor AMGMDS3 in a panel of tumor cell lines

**Figure 1:**
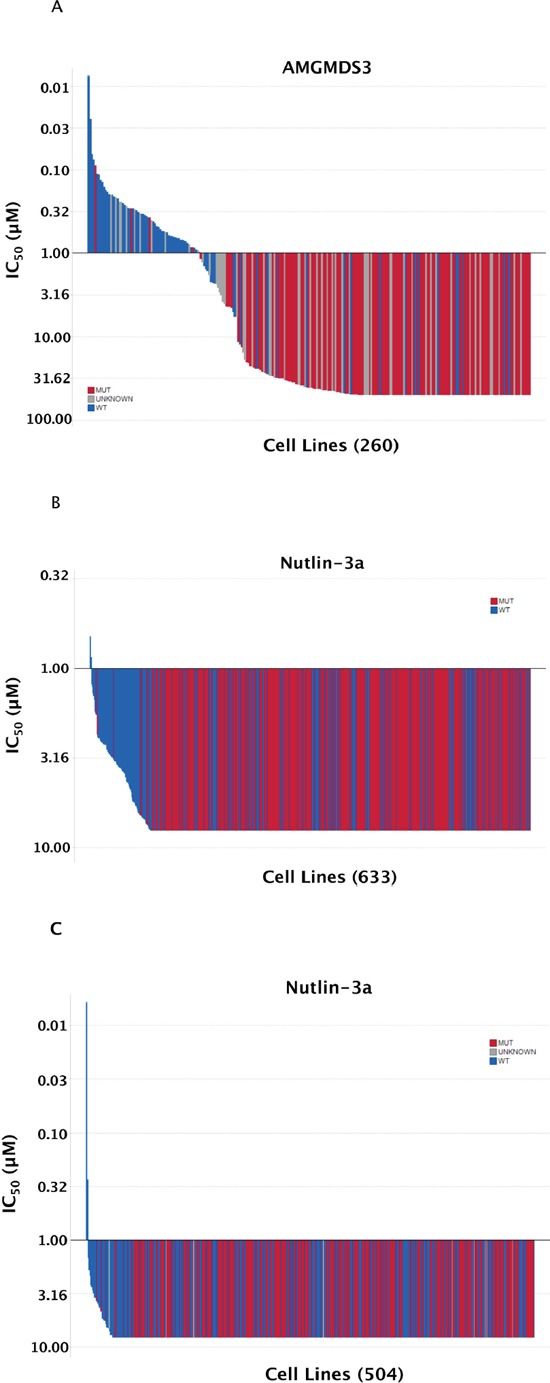
Sensitivity to MDM2 inhibition highly correlates with TP53 mutational status **(A)** The sensitivity to AMGMDS3 was profiled across a panel of 260 tumor cell lines in a 72-hour cell proliferation assay. The *TP53* mutational status of each cell line was annotated according to the data available in COSMIC (v44 release), http://www.sanger.ac.uk/cosmic [11, 28]. Similar representations of previously published nutlin-3a sensitivity data from **(B)** Garnett *et al*. [8] and **(C)** Barretina *et al*. [9] have been shown for comparison. The highest concentration of nutlin-3a tested by Garnett *et al*. and Barretina *et al*. was 8 μM.

As a first step towards identifying the determinants of sensitivity to MDM2 inhibition, a panel of 260 human tumor cell lines of diverse tissue origins was screened in a 72-hour cell proliferation assay. The effect of MDM2 inhibitor AMGMDS3 (Figure S1) on cell proliferation was determined by relative cell count as measured by nuclear staining, with IC_50_ values ranging from 0.01 μM to > 50 μM (Figure 1A, Table S1). In agreement with previous findings (plotted from published data in Figure 1B–1C; [8, 9]), sensitivity to MDM2 inhibition was highly correlated with p53 mutational status. This was a predictable result, as p53 mutations prevent p53 from activating transcriptional targets responsible for inducing cell cycle arrest and apoptosis. However, the correlation between p53 mutational status and sensitivity was not universal: some p53^Mutant^ cell lines appeared to be sensitive to MDM2 inhibition, while some p53^WT^ cell lines appeared to be insensitive. We suspected that some of these discrepancies might be related to misannotation or other confounding factors, and we therefore set out to comprehensively curate this cell line panel.

Twenty-six cell lines were removed from the dataset due to the poor growth characteristics of untreated cultures or high coefficient of variance between replicate untreated samples (Table S1). To authenticate the remaining cell lines, we extracted genomic DNA and performed genome-wide SNP analysis. We compared the resulting SNP profiles with those from the GlaxoSmithKline data repository (http://www.cabig.nci.nih.gov/community/caArray_GSKdata/; [10]) and Wellcome Trust Sanger Institute Cancer Genome Project (http://www.sanger.ac.uk/genetics/CGP; [11]). We determined that 5 cell lines had been misidentified and 22 cell lines were synonymous with one or more cell lines already represented in the panel (Table S1). These cell lines were excluded from further analysis.

### Functional inactivation of wildtype p53 by viral genes can affect proper assignment of p53 mutational status

The E6 protein from human papillomavirus (HPV) is known to bind p53 and promote its degradation via the ubiquitin pathway [12]. Proteins from DNA polyoma viruses SV40 (TAg) and adenovirus (E1B) also associate with p53 to form stable complexes (reviewed in [13]). While these virally-infected cell lines typically possess wildtype p53 alleles, they lack functional p53 protein. To determine whether any lines in the panel harbored viral DNA, we used PCR to screen their genomic DNA for HPV E6 (high-risk types 16, 18, 31, 33, and 45), SV40 large T antigen, and adenovirus E1B sequences (Table S2). Six lines were found to contain viral E6 DNA sequences from HPV16 (DoTc2 4510, SiHa, and engineered line RKO E6 [14]) or HPV18 (C-4 I, C-4 II, and HeLa). In addition, SV40 large T antigen sequence was detected in BPH1 and NCI-H295R. Adenovirus E1B sequence was not detected in any of the cell lines. All cell lines positive for the presence of viral DNA sequence were excluded from further analysis (Table S1).

### Genomic sequencing alone can determine p53 mutational status for many, but not all, cell lines

In order to establish the p53 status of the cell lines in the panel, exons 2–11 of the *TP53* gene, along with portions of the neighboring introns, were sequenced from genomic DNA samples extracted from each of the cell lines tested, with the exception of VCAP (sample unavailable). *TP53* sequence was determined for nearly all of the cell lines; the cell lines that failed sequencing for a subset of exons were annotated as deletion mutants (Table S1). Additionally, twenty-five cell lines were identified as p53^Mutant^/p53^WT^ heterozygotes by sequencing (Table S1) and were excluded from the dataset to avoid ambiguity.

We utilized the IARC *TP53* database to evaluate each of the sequenced missense mutations based on the comprehensive functional analysis of p53 mutant proteins performed by Kato *et al*. [15]. Nearly all of the mutations that we identified were annotated as inactivating. However, one amino acid substitution, Q331R in cell line 22Rv1, was shown by Kato *et al*. to be transcriptionally active. Therefore, this cell line was annotated as functionally wild-type and retained for further analysis.

**Figure 2:**
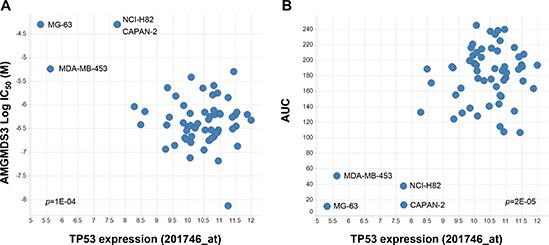
Preliminary linear regression association analysis indicates that low p53 expression correlates with insensitivity to MDM2 inhibition Scatter plots of **(A)** AMGMDS3 cell proliferation IC_50_ (*p* = 1E-04) or **(B)** integrated area under the dose response curve (*p* = 2E-05) versus *TP53* gene expression (201746_at; [10]) for the 62 cell lines with wildtype *TP53* genomic sequence profiled in this study. Further analysis revealed that CAPAN-2, MDA-MB-453, MG-63, and NCI-H82 were, in fact, p53^Mutant^ cell lines (see Figure 3).

**Figure 3:**
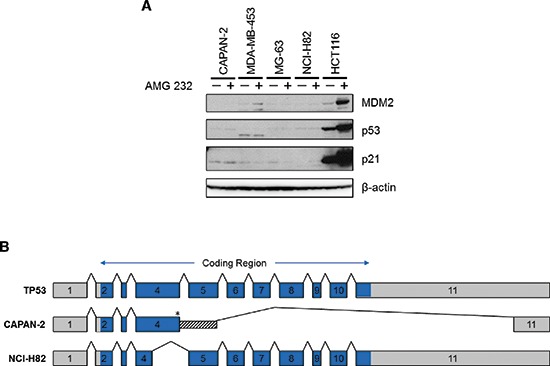
Four insensitive cell lines with wildtype TP53 genomic sequence are actually mutant **(A)** CAPAN-2, MDA-MB-453, MG-63, NCI-H82, and HCT116 cells were treated with either DMSO or 10 μM AMG 232 for 24 hours. Total protein lysates were collected, and immunoblot analysis of MDM2, p53, and p21 expression was performed. With the exception of HCT116, expression levels of these proteins were unaffected by MDM2 inhibition, suggesting that p53 was non-functional in these lines. Additionally, MDA-MB-453 lysates contained a faster migrating band, indicating truncation of p53 in that cell line. **(B)** Transcript sequencing of *TP53* cDNA generated from CAPAN-2 and NCI-H82 showed aberrantly spliced mRNA due to a silent G > T transversion in the last nucleotide of exon 4 (*, Thr125). In CAPAN-2, the resulting transcript included the first 271 bp of intron 4 spliced to the final 240 bp of exon 11, with deletion of all exons in between. In NCI-H82, the aberrant transcript deleted the last 200 bp of exon 4 (aa 59–125).

As part of the cell panel curation, we searched for drug sensitivity correlates that might reveal previously unrecognized confounders to the stratification analysis. Strikingly, we observed that the four least responsive p53^WT^ cell lines (CAPAN-2, MDA-MB-453, MG-63, and NCI-H82) also displayed the lowest expression levels of *TP53* transcript (Figure 2). Indeed, these 4 cell lines occupied a spatially distinct cluster in the plots of sensitivity vs. *TP53* expression. To further investigate p53 expression in these cell lines, immunoblot analysis was performed following 24 hours of treatment with MDM2 inhibitor AMG 232 [6]. HCT116, a p53^WT^ cell line that is sensitive to MDM2 inhibition, was used as a control in these experiments. As expected, AMG 232 treatment of HCT116 cells resulted in upregulation of MDM2 and p21 expression, as well as accumulation of p53 (Figure 3A). No such upregulation was seen in the other 4 cell lines, suggesting that p53 was non-functional in these lines. Additionally, in MDA-MB-453 cells, a band which migrated faster than the control was detected, indicative of a truncated mutant p53 protein, consistent with previously reported data (Figure 3A; [16]).

To investigate the mechanism(s) underlying the apparent p53 inactivation in CAPAN-2, MG-63, and NCI-H82, we sequenced *TP53* cDNA from these cell lines to determine whether or not there were mutations present in the transcript. *TP53* cDNA could not be isolated from MG-63 (data not shown). Since there were previously published data for MG-63 which demonstrated that *TP53* is rearranged within the first intron [17], this line was annotated as mutant. For CAPAN-2 and NCI-H82, *TP53* cDNA clones were generated and sequenced. Sequence alignment against the *TP53* cDNA reference sequence NM_000546 showed that a silent G > T mutation of the last nucleotide of exon 4 (Thr125Thr) affected splicing in both CAPAN-2 and NCI-H82 (Figure 3B), albeit differently. Interestingly, genomic DNA sequencing for *TP53* in CAPAN-2 and NCI-H82 detected the transversion, but it was not originally identified as a mutation in these samples since it was erroneously considered a SNP (rs55863639). T125T has been reported elsewhere as a mutation known to cause errors in splicing [18–20].

**Figure 4:**
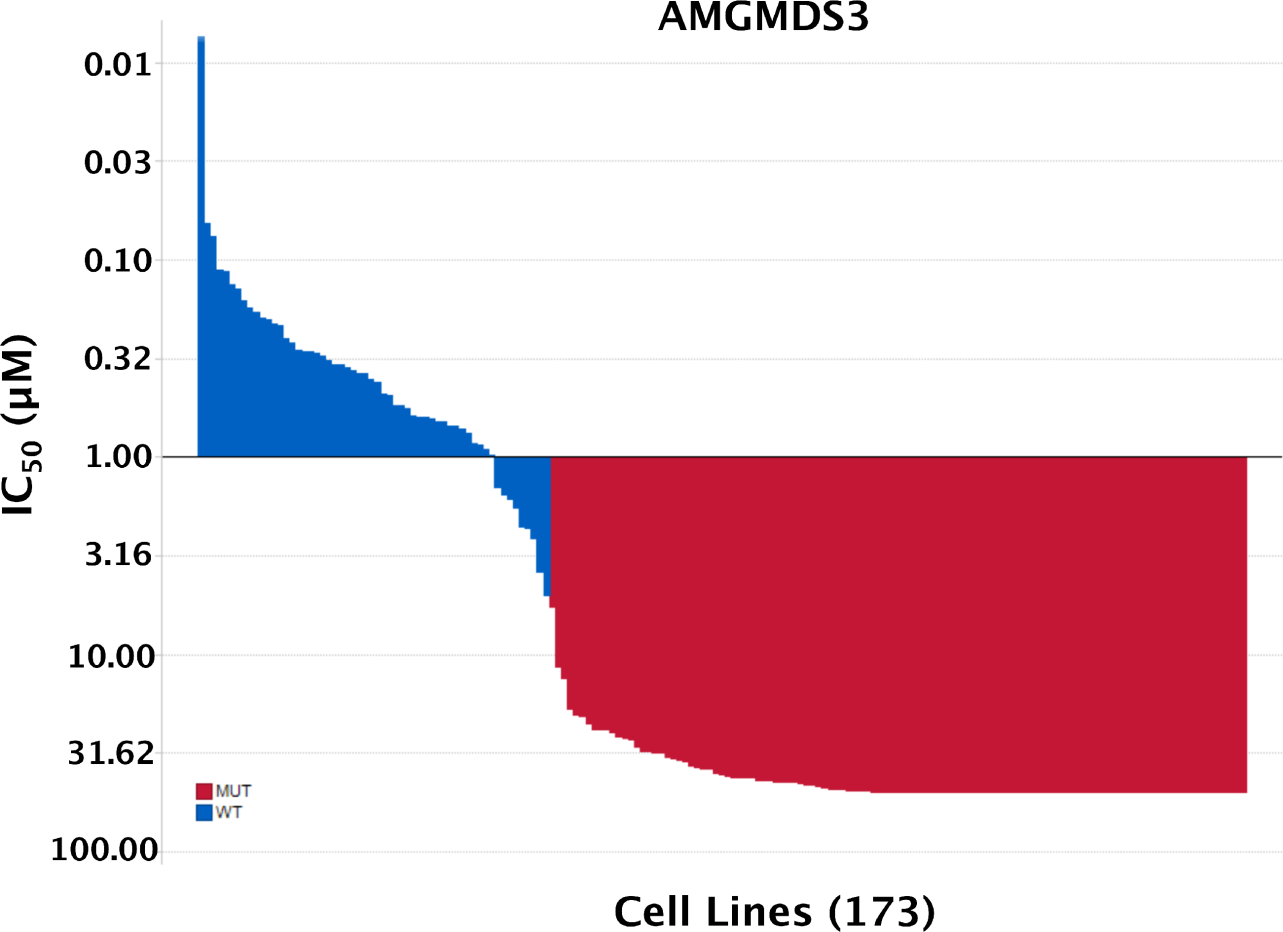
Sensitivity to MDM2 inhibition absolutely stratifies according to TP53 mutational status 260 cell lines were originally profiled for sensitivity to MDM2 inhibitor AMGMDS3. Cell lines were removed from the panel for the following reasons: poor growth/high variance of untreated samples (26), lack of genomic DNA sample (1), misidentification (5), redundancy (22), viral infection (8), heterozygous *TP53* mutation (25). The final curated set numbered 173 cell lines, of which 58 were p53^WT^ and 115 were p53^Mutant^.

With the correction of the annotation of the 4 low-expressing “p53^WT^” cell lines to p53^Mutant^, the final curated set numbered 173 cell lines (Table S1). Of these, 58 lines were p53^WT^ and 115 were p53^Mutant^. Sensitivity to MDM2 inhibition stratified absolutely according to p53 mutational status in the curated set (Figure 4).

We noted that the p53^WT^ cell lines as a group exhibited a wide range of IC_50_ values in response to MDM2 inhibition, and we therefore sought to identify additional predictors of sensitivity that might substratify these cell lines. We employed an ANOVA model which factored in tissue origin to identify genes whose expression appeared to be correlated with IC_50_ response (Table S3). The single most correlated gene from this model was then used to predict response in a logistic regression model. To evaluate the predictive model building process, we used leave-one-out (LOO) cross-validation and permutation testing [21]. For each permutation, the response labels were randomly shuffled within a given tissue, and then the LOO cross-validation was repeated. We then compared the original LOO cross-validation performance (% correct calls) to the empirical distribution of results with permuted data. The performance of the model fell at the 13^th^ percentile of all model results based on the shuffled data. We further addressed the issue of imbalance by restricting the initial LOO-cross-validation studies to only those tissues that had both responders and non-responders. In this case, the LOO performance fell at the 67^th^ percentile. The results from both analyses were no different than those expected by chance, indicating that the putative stratifiers identified in our initial analysis (Table S3) were unlikely to be predictive. Furthermore, within the set of confirmed p53^WT^ cell lines, we also evaluated associations between AMGMDS3 sensitivity and mutation data from 64 key cancer-associated genes in the Sanger COSMIC Cell Line Project [11]. We did not identify any significant associations between sensitivity to MDM2 inhibition and mutation of these genes (Table S4).

### *MDM2* amplification rates are likely lower than previously reported

It has been reported that p53^WT^ tumor cell lines that also harbor genetically amplified *MDM2* are particularly sensitive to MDM2 inhibitors [22]. To determine whether this enhanced sensitivity might occur clinically, it will be critical to accurately identify which patients' tumors harbor *bona fide MDM2* amplification. A frequently cited literature review reporting aggregate data compiled from multiple studies suggested that pan-cancer *MDM2* amplification rates may be as high as 7% [23], but the underlying studies used a varied array of methodologies and employed no standardized amplification copy number cutoffs. Furthermore, the reported overall amplification rate suffered from ascertainment bias, as a disproportionate number of the studies focused on tumor types previously reported to harbor high rates of *MDM2* amplification.

**Figure 5:**
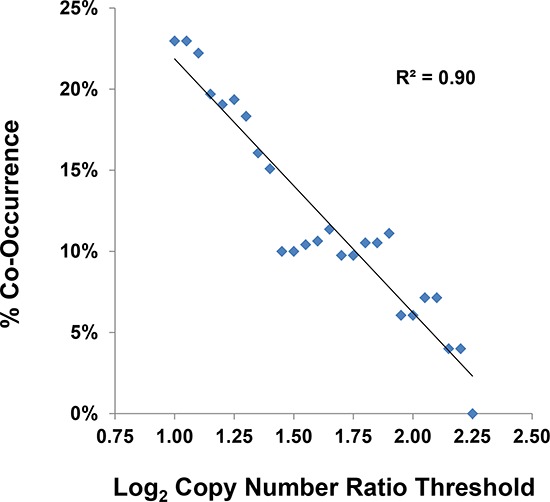
The threshold of functionally relevant MDM2 amplification is ≥ 9.5 copies Co-occurrence of *MDM2* amplification and *TP53* mutation was analyzed using data collected by the TCGA Research Network [4] for 3856 human tumor samples. Assuming that there was no selective advantage for tumor cells to contain both inactivating mutations of the p53 pathway, the copy number threshold associated with mutual exclusivity between *MDM2* amplification and *TP53* mutation was a log_2_ ratio ≥ 2.25. See also Table S5.

To better define the frequency of *MDM2* amplification in human cancer, we took advantage of the observation that *MDM2* amplification and *TP53* mutation are mutually exclusive phenomena [24]. This finding implies that there is no selective advantage for a tumor to have two inactivating mutations of the p53 pathway. In our analysis, we assumed that the definitive cutoff for functionally relevant *MDM2* amplification was the copy number associated with mutual exclusivity between *MDM2* amplification and *TP53* mutation. Using Oncomine NGS PowerTools (Life Technologies) to visualize the data generated by the TCGA Research Network [4], we applied this hypothesis to a pan-cancer analysis of 3856 TCGA tumor samples with both copy number and sequence data. After correcting for functional *TP53* mutations [15], we observed that co-occurrence of *MDM2* amplification and *TP53* mutation decreased as the cutoff value for *MDM2* copy number increased (Figure 5 and Table S5; R^2^ = 0.9). The incidence of co-occurrence for these two alterations fell to zero at *MDM2* copy number ≥ 9.5 (log_2_ CN ratio ≥ 2.25), suggesting that *MDM2* amplification in this range was capable of functionally inactivating the p53 pathway. Applying this copy number cutoff to all tumor types represented in TCGA (Table 1) yielded *MDM2* amplification rates far lower than those published previously [23], providing a more rationally defined threshold for enrolling patients into clinical trials designed to test whether *MDM2* amplification affords enhanced sensitivity to MDM2 inhibition.

**Table 1:** Annual U.S. incidence of *MDM2*-amplified tumors

**Tumor Type**	***MDM2* Amplification^†^(TCGA^‡^)**	**Annual Incidence^[30]^ (U.S.)**	**Annual U.S. Incidence with *MDM2* Amplification**
Liposarcoma	64% (9/14)	5,000^[31]^	3,200
Glioblastoma Multiforme	7.3% (41/562)	11,130^[32]^	812
Bladder Urothelial Carcinoma	2.6% (4/152)	74,690	1,942
Stomach Adenocarcinoma	1.5% (4/273)	22,220	333
Lung Adenocarcinoma	0.9% (4/437)	66,700^[33]^	600
Skin Cutaneous Melanoma	0.7% (2/273)	76,100	532
Brain Lower Grade Glioma	0.5% (1/220)	3,000^[32, 34]^	15
Breast Invasive Carcinoma	0.3% (3/926)	235,030	706
Ovarian Serous Cystadenocarcinoma	0.2% (1/576)	13,190^[35]^	26
Uterine Corpus Endometrial Carcinoma	0% (0/493)	52,630	-
Lung Squamous Cell Carcinoma	0% (0/387)	57,170^[33]^	-
Head and Neck Squamous Cell Carcinoma	0% (0/337)	42,440	-
Kidney Renal Papillary Cell Carcinoma	0% (0/117)	6,400^[36]^	-
**TOTAL**			**8,166**

† *MDM2* Log_2_ CN Ratio Cutoff = 2.25.

‡ The occurrence of *MDM2* amplification was derived from tumor sample data generated by the TCGA Research Network [4].

## DISCUSSION

While MDM2 inhibitors hold great potential as cancer therapeutics, the ability to predict which tumors will respond to these agents will be critical to realizing this promise. In principle, all p53^WT^ tumors have the capacity to undergo cell cycle arrest upon p53 activation, and many such tumors are also expected to undergo apoptosis. In contrast, p53^Mutant^ tumors would not be predicted to respond to MDM2 inhibition. Cell line sensitivity studies using MDM2 inhibitors have trended in support of these predictions, but there are numerous examples of cell lines that appear to defy these expectations.

To better understand the nature of these exceptions, we screened a panel of 260 cell lines with a potent and selective MDM2 inhibitor (AMGMDS3) to assess effects on viability. The resulting IC_50_ values ranged over approximately 3 orders of magnitude (Figure 1A), where the least sensitive cell lines had IC_50_ values ≥ 50 μM (the highest concentration tested). By comparison, analogous screens performed by Garnett *et al*. [8] and Barretina *et al*. [9] using the MDM2 inhibitor, nutlin-3a, resulted in IC_50_ values spanning only about a 10-fold range across the vast majority of the lines profiled (Figure 1B and 1C, respectively). The larger dynamic range afforded by AMGMDS3 over nutlin-3a is likely responsible for the clearer separation of p53^WT^ and p53^Mutant^ cell lines by sensitivity. Nonetheless, even with the larger dynamic range, the initial correlation between sensitivity and p53 mutational status in our screen was not absolute. To eliminate factors potentially confounding our sensitivity analysis, we extensively curated this panel, removing cell lines that were (1) misidentified, (2) heterozygous for p53 mutation, or (3) harboring viral gene sequences known to inactivate p53. Additionally, we corrected p53 mutational status when it was misannotated and determined p53 mutational status when it was unknown. We also removed cell lines with poor growth characteristics or high coefficients of variance between replicate untreated samples. Following curation of the panel, the apparent discrepancies between sensitivity prediction and experimental observation were eliminated, revealing a perfect correlation between p53 mutational status and MDM2 inhibitor responsiveness. Based on these results, only patients with p53^WT^ tumors are being enrolled into clinical studies involving Amgen's MDM2 inhibitor, AMG 232.

While all tumor cell lines containing functional p53 responded to MDM2 inhibition in our screen, these lines still exhibited a 500-fold range in IC_50_ values from least to most sensitive. We attempted to identify the determinants of this heterogeneity by searching for genetic or expression predictors of sensitivity. Although a small number of gene expression variables were initially found to display a potential correlation between expression and sensitivity (Table S3), the fold change in expression of these genes was marginal (range 0.64 – 1.61), and the logistic models built on this gene expression dataset were not significant. Further, there were no significant associations between response and mutational status of 64 key cancer-associated genes (Table S4). The failure to successfully predict response may have been due to the small number of p53^WT^ cell lines under examination or the challenges in finding such correlates in cell lines from a diverse set of tumor origins.

This report has focused on the use of MDM2 inhibitors to reduce the viability of p53^WT^ tumor cells. However, this is not the only context in which an MDM2 inhibitor might have clinical utility. An alternative approach has been proposed which involves the combined use of an MDM2 inhibitor with a mitotic inhibitor to treat patients harboring p53^Mutant^ tumors [25, 26]. Under this scenario, the MDM2 inhibitor would selectively induce reversible G1 and/or G2 cell cycle arrest in most host tissues, thus protecting them from the cytotoxic effects of chemotherapy targeting cells in M-phase. In contrast, the p53^Mutant^ tumor cells, unaffected by MDM2 inhibition, would continue to cycle through M-phase, during which they would be susceptible to the mitotic inhibitor. In this therapeutic approach, the determinants of differential tumor response would be defined by the sensitivity of each cancer to the mitotic inhibitor, rather than to the MDM2 inhibitor.

It has been reported that the *MDM2*-amplified subset of p53^WT^ tumors might be particularly sensitive to MDM2 inhibition [22]. Due to the very limited number of such cell lines in the public domain, we did not attempt to address this question in our cell panel. Instead, we chose to pursue an approach that might facilitate our ability to test this hypothesis clinically. We assumed that *MDM2* amplification and *TP53* mutation were mutually exclusive means for a tumor to inactivate the p53 pathway, as has been borne out in sarcomas [24]. With this hypothesis in mind, we mined the human tumor data generated by the TCGA Research Network [4] and identified a rationally derived copy number cutoff for assignment of functionally relevant *MDM2* amplification. Applying this cutoff across all tumor samples within TCGA resulted in a pan-cancer *MDM2* amplification rate far lower than previously published [23]. Upcoming AMG 232 clinical studies will use the same threshold to test whether *MDM2*-amplified tumors are especially sensitive to MDM2 inhibition.

## METHODS

### Cell lines

Cell lines were purchased from American Type Culture Collection (ATCC), German Collection of Microorganisms and Cell Cultures (DSMZ), and Japanese Collection of Research Bioresources (JCRB). All cell lines were passaged less than 1 month prior to banking and experimentation. Unless recommended otherwise, cell lines were cultured in RPMI 1640 medium supplemented with 10% fetal bovine serum, 1 mM sodium pyruvate, 2 mM L-alanine, and 2 mM L-glutamine. For authentication of cell lines at ATCC, DSMZ and JCRB, short-tandem repeat DNA typing was used.

Further authentication of a subset of cell lines was performed by Expression Analysis using the Affymetrix Genome-Wide Human SNP array 6.0. SNP genotype determination was performed using ArrayStudio. At each available SNP location, comparison was made between each of the tested samples and publically available SNP data from GlaxoSmithKline (http://www.cabig.nci.nih.gov/community/caArray_GSKdata/; [10]) or from the Wellcome Trust Sanger Institute Cancer Genome Project (http://www.sanger.ac.uk/genetics/CGP/; [11]). The total number of locations with a known genotype and the number of matched locations were tallied, and the percent genotype match was calculated for each sample. From this analysis, the best matched cell line from GSK or Wellcome Trust Sanger Institute was identified. In addition, a separate analysis was performed in the same manner, comparing the GSK SNP genotype data for all cell lines in the panel to the Wellcome Trust Sanger Institute CGP SNP data in order to identify any synonymous or misidentified cell lines.

### Cell proliferation assay

Inhibition of cell proliferation was measured by Eurofins Panlabs (formerly Ricerca Biosciences). Cells were seeded into 384-well plates. After 24 hours, a 10-point titration of MDM2 inhibitor was added to the wells in a final DMSO concentration of 0.1%. After 72-hour treatment, cells were fixed and stained with dye to allow visualization of nuclei. Automated fluorescence microscopy was carried out using a GE Healthcare IN Cell Analyzer 1000, and images were collected with a 4X objective.

Cell proliferation was measured by incorporation of nuclear dye to determine the relative cell count. To calculate the effect of compound treatment on cell proliferation, the data were transformed to percent of control (POC) using the following formula: POC = relative cell count (compound wells)/relative cell count (vehicle wells) × 100. IC_50_ values were derived using nonlinear regression to fit data to a sigmoidal 4-point, 4-parameter one-site dose response model, where: y (fit) = A + [(B − A)/(1 + ((C/x) ^ D))]. Relative cell count IC_50_ was the inhibitor concentration that produced 50% of the cell proliferation inhibitory response or 50% cytotoxicity level, relative to DMSO control.

### Genomic analysis of TP53

Genomic DNA was extracted from frozen cell pellets of each tumor cell line according to the DNeasy 96 kit protocol (QIAGEN) and sequenced for the presence of mutations in *TP53*, either using 454 sequencing (Roche Diagnostics) or WAVE^®^ Mutation Detection System and SURVEYOR^®^ Nuclease technology (Transgenomic, Inc.; [27]).

Additional data regarding the p53 mutational status of tumor cell lines in the panel were obtained from the Wellcome Trust Sanger Institute Catalogue of Somatic Mutations in Cancer (COSMIC v44–62 releases), http://www.sanger.ac.uk/cosmic [11, 28]; the IARC *TP53* database (version R15), p53.iarc.fr/TP53GeneVariations.aspx [29], and the *TP53* Mutation Database, http://www.p53.fr [18].

### Quantitative PCR screening for p53-inactivating viral DNA sequences

Primers to detect viral DNA sequences for human papillomavirus E6 from high-risk variants 16, 18, 31, 33, and 45, as well as simian virus 40 large T antigen and adenovirus E1B were designed using Primer Express software and synthesized by Integrated DNA Technologies (Table S2). Genomic DNA samples from human tumor cell lines were screened by quantitative RT-PCR using an Applied Biosystems Prism 7900HT instrument.

### Sequencing of TP53 transcript

Total RNA was isolated from human tumor cell lines according to the RNeasy Mini Kit protocol (QIAGEN). 3′RACE-ready cDNA was generated according to the SMARTer RACE cDNA Amplification Kit protocol (Clontech). 3′ RACE PCR, followed by additional nested PCR, was performed to amplify *TP53* cDNA sequence from each of the samples. PCR products were electrophoresed on 1% agarose gels, and the desired PCR products were isolated, purified, cloned, and sequenced. Sequences from the cDNA clones were aligned against the *TP53* cDNA reference sequence NM_000546 using Vector NTI^®^ software (Life Technologies).

### Immunoblot analysis

Cells were seeded at subconfluent densities in 6-well plates and incubated overnight at 37°C and 5% CO_2_. The following day, either DMSO or AMG 232 was added to appropriate wells at a final concentration of 0.1% DMSO or 10 μM, respectively. After incubation for 24 hours at 37°C and 5% CO_2_, protein lysates were collected, electrophoresed on 10% Bis-Tris NuPAGE gels (Life Technologies), and transferred to Invitrolon PVDF membranes (Life Technologies). Following transfer, the membranes were blocked and incubated with primary antibodies against p53 (DO-1; Calbiochem), MDM2 (BD Pharmingen), p21 (R&D Systems), or β-actin-HRP (Sigma). For detection, the membranes were incubated with species-specific secondary antibodies conjugated to HRP. Luminescent signal was developed using ECL Plus reagent (GE Healthcare) and the membranes were exposed to film.

### Association analysis

The vast majority of the gene expression data was obtained from the GlaxoSmithKline Cancer Cell Line Genomic Profiling dataset [10]. For cell lines without publicly available data, analogous data were obtained from Eurofins Panlabs (formerly Ricerca Biosciences). Gene expression data were generated using the Affymetrix HG-U133 Plus 2.0 microarray platform. RMA-normalized gene expression data from Greshock *et al*. [10] and from Eurofins Panlabs were combined using a per probe set linear batch correction based on 20 cell lines profiled in both data sets.

Association analyses were performed to attempt to identify additional predictors of sensitivity. Response to MDM2 inhibition was categorized as true if the IC_50_ for a cell line was in the lower quartile of IC_50_ values among all cell lines; otherwise it was set to false. An ANOVA model including a factor for tumor origin was used as a feature selection step to identify genes whose expression were highly correlated with IC_50_ response. The single most correlated gene from the feature selection step was used in a logistic regression model to predict response given the gene expression value and tissue source of a new cell line. If the model-based probability of response was predicted to be greater than 0.5, the new cell line was classified as a responder; otherwise it was classified as a non-responder. The leave-one-out (LOO) cross-validation procedure recommended by Simon *et al*. [21] was used to estimate the performance of the entire model building process, including feature selection. A single sample was left out, and the remainder was used to identify a gene expression variable and to build a predictive model. The predictive model was used to predict response (true/false) of the sample that was left out. The process was repeated, leaving each sample out in turn. At the end of the LOO cross-validation, the predictions were compared to known results. The performance of the LOO cross-validation was evaluated based on permutation testing. For each permutation, the response labels were randomly shuffled within a given tissue, and then the LOO cross-validation was repeated. We then compared the original LOO cross-validation performance (% correct calls) to the empirical distribution of results with permuted data. We further addressed the issue of imbalance by restricting the initial LOO-cross-validation studies to only those tissues that had both responders and non-responders.

Mutation data were collected from the Wellcome Trust Sanger Institute COSMIC Cell Line Project (http://www.cancer.sanger.ac.uk/cancergenome/projects/cell_lines/; v61 release) [11]. The mutation status of 64 key cancer-associated genes was available for 51 of the 58 p53^WT^ cell lines in the panel. There were 25 genes for which at least one cell line was called mutant. We evaluated associations between response to MDM2 inhibition and mutation status using the “aov” function in R. Test *p*-values were adjusted using the R “p.adjust” function with method = “BH”.

## SUPPLEMENTARY FIGURE AND TABLES




