# Supplementary material for: Identifying the determinants of response to MDM2 inhibition
Source: Oncotarget. 2015 Feb 3;6(10):7701–12. doi: 10.18632/oncotarget.3116 (PMC4480710; doi:10.18632/oncotarget.3116)
Supplement: Supplementary file 1 [file oncotarget-06-7701-s001.pdf]

SUPPLEMENTARY FIGURE AND TABLES

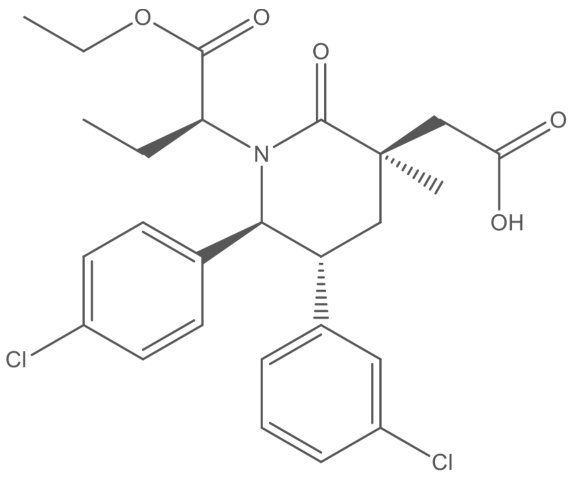

HTRF (no serum) IC_{50}= 2.4 ± 0.6 nM
HTRF (15% human serum) IC_{50}= 20.3 ± 3.2 nM

Supplementary Figure S1: Related to Table S1. AMGMDS3. Experimental details of the HTRF *in vitro* potency assays can be found in the supporting information of Ref. [6]

Supplementary Table S1: Related to Figures 1A, 2, 4. 260 cell lines profiled for sensitivity to AMGMDS3 with annotated *TP53* mutational status. TP53 status (column G) was obtained from the Wellcome Trust Sanger Institute Catalogue of Somatic Mutations in Cancer (COSMIC v44–62 releases), http://www.sanger.ac.uk/cosmic [11, 28].

Supplementary Table S2: Primer sets for quantitative PCR detection of viral DNA sequences

Target	Accession	Primer	Sequence
**Adenovirus E1B**	AY601635	F	GACAGGGCCTCTCAGATGCT
		R	TGGCTACGTGAATGGTCTTCAG
		P	TGCTCGGACGGCAACTGTCACCT
**HPV16 E6**	K02718.1	F	ACCGTTGTGTGATTTGTTAATTAGGT
		R	GCTTTTTGTCCAGATGTCTTTGC
		P	AACTGTCAAAAGCCACTGTGTCCTGAAGAAA
**HPV18 E6**	AY262282.1	F	AAAACGACGATTTCACAACATAGC
		R	CTGTCGTGCTCGGTTGCA
		P	CACTATAGAGGCCAGTGCCATTCGT
**HPV31 E6**	J04353.1	F	GACCTCGGAAATTGCATGAAC
		R	CCTTTGCAGTAGACACAATTCAATCT
		P	AGCTCGGCATTGGAAATACCCTACGATGA
**HPV33 E6**	EU918766.1	F	GATTTGTGCCAAGCATTGGA
		R	GATCGTTGCAAAGGGTTTCTG
		P	ACAACTATACACAACATTGAACTACAGTGCGTGGAA
**HPV45 E6**	Y13218.1	F	GCCTGCGGTGCCAGAA
		R	CTGTGGAATCTTCGTTTGTCCTT
		P	CATTGAACCCAGCAGAAAAACGTAGACACCT
**SV40 Large T**	J02400.1	F	TGAGAGTCAGCAGTAGCCTCATCA
		R	GTGGAATGCCTTTAATGAGGAAA
		P	CCTGTTTTGCTCAGAAGAAATGCCATCTAGTG

F, forward primer; R, reverse primer; P, probe. Probes were labeled with 5’-FAM (6-carboxyfluorescein) and 3’-TAMRA (carboxytetramethylrhodamine).

Supplementary Table S3: Gene expression associated with varying sensitivity to AMGMDS3 (adjusting for tissue-specific expression)

Probe	Gene Symbol	Description	Fold Change (95% CI)	*p*-value
231948_s_at	UBE2F	ubiquitin-conjugating enzyme E2F (putative)	0.86 (0.82–0.91)	2.60E-06
201538_s_at	DUSP3	dual specificity phosphatase 3 (vaccinia virus phosphatase VH1-related)	0.86 (0.80–0.92)	5.32E-05
241709_s_at	DOCK1	dedicator of cytokinesis 1	0.87 (0.81–0.93)	8.83E-05
201199_s_at	PSMD1	proteasome (prosome, macropain) 26S subunit, non-ATPase, 1	0.91 (0.88–0.95)	4.19E-05
205680_at	MMP10	matrix metallopeptidase 10 (stromelysin 2)	0.71 (0.60–0.83)	1.33E-04
212311_at	KIAA0746	KIAA0746 protein	0.73 (0.62–0.86)	3.35E-04
202345_s_at	FABP5	fatty acid binding protein 5 (psoriasis-associated)	1.61 (1.20–2.15)	2.24E-03
219049_at	CHGN	chondroitin beta1,4 N-acetylgalactosaminyltransferase	1.42 (1.15–1.76)	1.61E-03
201744_s_at	LUM	lumican	0.64 (0.44–0.94)	2.29E-02

The predictive model building process was evaluated by leave-one-out cross-validation and permutation testing [21]. The performance of all model results based on permuted data indicated that the results reported in this table were not different from those expected by chance.

Supplementary Table S4: Association analysis of gene mutation with varying sensitivity to AMGMDS3

Gene Symbol	*p*-value	Adjusted *p*-value
CDKN2A	0.10	0.95
CDKN2ap14	0.19	0.95
VHL	0.19	0.95
NOTCH1	0.34	0.95
KDM6A	0.36	0.95
BRAF	0.45	0.95
PTEN	0.52	0.95
TSC1	0.62	0.95
STK11	0.66	0.95
CDH1	0.67	0.95
SMARCA4	0.68	0.95
PIK3CA	0.71	0.95
SETD2	0.73	0.95
NF2	0.76	0.95
IDH1	0.79	0.95
FBXW7	0.80	0.95
NF1	0.80	0.95
CDKN2C	0.81	0.95
EGFR	0.82	0.95
MSH2	0.82	0.95
SF3B1	0.84	0.95
TP53	0.89	0.95
NRAS	0.90	0.95
CTNNB1	0.93	0.95
KRAS	0.95	0.95

Mutation data for 64 key cancer genes in 51 of the 58 $p53^{WT}$ cell lines in our panel were collected from the Sanger COSMIC Cell Line Project (v61 release; cancer.sanger.ac.uk/cancergenome/projects/cell_lines/) [11]. Associations were evaluated between response to MDM2 inhibition and mutation status.

Supplementary Table S5: Related to Figure 5. Co-occurrence of *MDM2* amplification and *TP53* mutation across 3856 TCGA tumor samples

Log_2 Copy Number Ratio	# of Samples with *MDM2* CN ≥ Cutoff	# of Samples with *TP53* Mutation and *MDM2* CN ≥ Cutoff	% Co-occurrence
1.00	74	17	23%
1.05	74	17	23%
1.10	72	16	22%
1.15	66	13	20%
1.20	63	12	19%
1.25	62	12	19%
1.30	60	11	18%
1.35	56	9	16%
1.40	53	8	15%
1.45	50	5	10%
1.50	50	5	10%
1.55	48	5	10%
1.60	47	5	11%
1.65	44	5	11%
1.70	41	4	10%
1.75	41	4	10%
1.80	38	4	11%
1.85	38	4	11%
1.90	36	4	11%
1.95	33	2	6%
2.00	33	2	6%
2.05	28	2	7%
2.10	28	2	7%
2.15	25	1	4%
2.20	25	1	4%
2.25	23	0	0%

Based on data generated by the TCGA Research Network [4] and analyzed using Oncomine NGS PowerTools (Life Technologies).
